# Clinical Characteristics and Mortality Risks Among Patients With Culture-Proven Coccidioidomycosis Who Are Critically Ill: A Multicenter Study in an Endemic Region

**DOI:** 10.1093/ofid/ofae454

**Published:** 2024-08-07

**Authors:** James Lim, Ashley M Scott, Rebecca Wig, Rachel V Tan, Emily R Harnois, Tirdad T Zangeneh, Mohanad M Al-Obaidi

**Affiliations:** Department of Internal Medicine, University of Arizona College of Medicine, Tucson, Arizona, USA; Division of Infectious Diseases, University of Arizona College of Medicine, Tucson, Arizona, USA; Department of Internal Medicine, University of Arizona College of Medicine, Tucson, Arizona, USA; Department of Internal Medicine, University of Arizona College of Medicine, Tucson, Arizona, USA; Department of Internal Medicine, University of Arizona College of Medicine, Tucson, Arizona, USA; Department of Internal Medicine, University of Arizona College of Medicine, Tucson, Arizona, USA; Department of Internal Medicine, University of Arizona College of Medicine, Tucson, Arizona, USA; Division of Infectious Diseases, University of Arizona College of Medicine, Tucson, Arizona, USA; Valley Fever Center for Excellence, University of Arizona College of Medicine, Tucson, Arizona, USA; Department of Internal Medicine, University of Arizona College of Medicine, Tucson, Arizona, USA; Division of Infectious Diseases, University of Arizona College of Medicine, Tucson, Arizona, USA; Valley Fever Center for Excellence, University of Arizona College of Medicine, Tucson, Arizona, USA

**Keywords:** antifungals, coccidioidomycosis, intensive care unit

## Abstract

**Background:**

Coccidioidomycosis is an endemic mycosis in the southwestern United States. While most infections are mild, severe cases can be devastating. We aimed to describe the clinical characteristics and mortality risks of patients in the intensive care unit (ICU) with culture-proven coccidioidomycosis.

**Methods:**

We performed a retrospective chart review of patients in the ICU with positive *Coccidioides* spp culture in a large health care system in Arizona between 1 October 2017 and 1 July 2022. All data were entered into REDCap.

**Results:**

An overall 145 patients were identified and included. The median age was 51 years, with the majority male (69%) and non-Hispanic White (39%). Most patients (n = 104, 72%) had pulmonary coccidioidomycosis, and 41 had extrapulmonary disease (17 meningitis, 13 fungemia, 10 musculoskeletal disease, and 4 pericardial or aortic involvement). Seventy patients (48%) died during hospitalization, and most (91%) received antifungal therapy during hospitalization. In the multivariate logistic regression model, age ≥60 years (odds ratio [OR], 7.0; 95% CI, 2.6–18.8), cirrhosis (OR, 13.1; 95% CI, 1.6–108.8), and mechanical ventilation or vasopressor support (OR, 15.4; 95% CI, 3.9–59.6) were independently associated with increased all-cause mortality, but pre-ICU antifungal use had a statistically insignificant mortality risk association (OR, 0.5; 95% CI, .2–1.2).

**Conclusions:**

In our study of patients in the ICU with coccidioidomycosis and multiple comorbidities, the mortality rate was high. Older age, cirrhosis, and mechanical ventilation or vasopressor support were significantly associated with high mortality. Future studies are recommended to evaluate those risk factors and the efficacy of rapid diagnosis and early therapy in patients at high risk.

## Teaser

Coccidioidomycosis in patients hospitalized in the intensive care unit are at increased risk of death. While antifungal use did not affect mortality outcome, older age, vasopressor or mechanical ventilation support, and liver cirrhosis were associated with increased mortality.

Coccidioidomycosis is a fungal infection caused by *Coccidioides immitis* and *Coccidioides posadasii* [1]. These species are endemic to the southwestern United States, predominantly Arizona and California [1, 2]. However, this geographic distribution may be wider, and it is suggested that it is likely to expand due to climate change [3, 4].

About two-thirds of coccidioidomycosis cases are associated with asymptomatic or mild respiratory symptoms [5, 6]. While most cases present as pulmonary disease, 1% to 3% present as a disseminated infection, nearly involving any organ [6]. Despite current knowledge, coccidioidomycosis diagnosis can be missed, even in the endemic regions, as pulmonary disease may account for almost a third of community-acquired pneumonia cases in endemic regions [7]. Moreover, coccidioidomycosis has been shown to account for most of the cases in Arizona presenting with a miliary pattern on chest imaging [8].

The diagnosis of coccidioidomycosis remains difficult and relies heavily on serologic tests, which have varying sensitivities and specificities. Individuals require a functioning immune system to mount a response; therefore, it can take weeks to seroconvert, and some patients who are immunosuppressed may not develop positive serology [1, 9–11]. Antigenic tests, such as the more specific coccidioidal antigen and less specific (1-3)-β-d-glucan, can be used for diagnosis, and both can be performed on blood and cerebrospinal fluid samples, with each having high sensitivity in cerebrospinal fluid to diagnose central nervous system infection [11, 12]. Definitive diagnosis can be made by isolating *Coccidioides* spp in culture or identifying spherules by histopathology in specimens [1, 10].

Some factors associated with severe or disseminated infection include Black race, Filipino ethnicity, older age, diabetes mellitus, pregnancy, and immunosuppression [6, 13–15]. Severe coccidioidomycosis can result in hospitalization and death, and historical data from Veterans Affairs–Armed Forces showed that the mortality rate among patients with disseminated infection was 25% [16]. Similarly, Jenks et al reported a 20% mortality among a cohort of hospitalized patients with positive *Coccidioides* spp cultures [17]. However, data are limited on patients who are critically ill with severe and/or disseminated coccidioidomycosis, with a recent analysis of data from the Agency for Healthcare Research and Quality reporting a mortality rate of 33% among patients admitted with coccidioidomycosis and requiring mechanical ventilation [18]. Moreover, among patients with coccidioidomycosis-associated acute respiratory distress syndrome, there was a 90-day mortality rate of 15% [19]. Yet, both studies included probable cases of coccidioidomycosis, diagnosed by serologic testing. We aimed to describe clinical characteristics and evaluate the risk factors for mortality among patients in the intensive care unit (ICU) with culture-proven coccidioidomycosis.

## METHODS

### Data Collection

We included nonpregnant patients aged ≥18 years who were hospitalized at a large multicenter health care system in Arizona from 1 October 2017 through 1 July 2022, requiring ICU admission with a positive *Coccidioides* species culture. Demographic data such as age, race, ethnicity, and gender were collected. Race was defined as White, Black, Asian/Pacific Islander, and others if the patient identified as 2 or more races or Native American. Ethnicity was defined as Hispanic or not-Hispanic. Patients admitted electively for surgical procedures requiring a brief ICU stay were excluded. Clinical data were collected regarding the presentation of coccidioidomycosis infection, antifungal therapy use, and *Coccidioides* serologic tests (enzyme immunoassay, immunodiffusion, or complement fixation). Any positive result within 4 weeks from positive culture was considered positive. Also, data were collected regarding the presence of other systemic infections (including COVID-19), the need for mechanical ventilation or vasopressor support, and other underlying comorbidities. The administration of any dose or duration of antifungals active against coccidioidomycosis prior to ICU admission was defined as pre-ICU antifungal for data analysis. Data were retrospectively extracted from the charts and entered into REDCap. The primary aim was to describe the clinical characteristics of hospitalized patients in the ICU with coccidioidomycosis. The secondary aim was to evaluate the treatment and risk factors associated with all-cause mortality at the end of hospitalization. Survival was defined as discharge from a hospital other than discharge to hospice or death.

### Statistical Analysis

Descriptive statistics were used to evaluate the underlying characteristics of the patients. The chi-square test was used to evaluate categorical variables, and the Kruskal-Wallis test was used to evaluate continuous variables between 2 groups. Univariate and multivariate logistic regressions were performed to evaluate each risk factor's association with all-cause mortality. Covariates that had a statistically significant association in the univariate analysis were included in the multivariate logistic regression. Antifungal therapy was evaluated in relation to ICU admission by evaluating the dichotomized variable of pre-ICU admission antifungal therapy on the all-cause mortality outcome after adjusting for other risk factors that had a *P* value <.2. Variables showing collinearity with pre-ICU antifungal therapy were not included in the multivariate model (ie, pre-ICU coccidioidomycosis diagnosis and history of coccidioidomycosis before admission). All performed statistics were 2-tailed tests with *P* values <.05 considered statistically significant. All statistical analysis was conducted with Stata IC version 16.1 (StataCorp).

### Ethical Statement

The study was reviewed and approved by the institutional review board of the University of Arizona College of Medicine and in accordance with the Belmont report. Patient consent was waived because the study was deemed below the minimal risk.

## RESULTS

### Demographics and Clinical Characteristics

Over the study period, there were 367 hospitalized patients with positive *Coccidioides* spp culture. Of those, 145 were admitted to the ICU and met the study inclusion criteria for final analysis. Demographics and clinical characteristics are depicted in Table 1. The median age was 51 years (range, 19–82); 100 patients were male (69%); and 56 were non-Hispanic White (39%). The majority of patients (n = 104, 72%) had only pulmonary coccidioidomycosis. Of the 41 (28%) with extrapulmonary disease, 17 (12%) were diagnosed with meningitis, 13 (9%) with fungemia, 10 (7%) with musculoskeletal disease, and 4 (3%) with pericardial or aortic involvement. There were 34 (23%) patients with a history of coccidioidomycosis before admission, with 5 patients not taking antifungal therapy before admission (2 stopped after thoracic surgical resection, and 3 had either an adverse reaction to azoles or reported nonadherence).

**Table 1.** Demographics and Clinical Characteristics of Patients With Coccidioidomycosis Who Required Intensive Care (n = 145)

	No. (%)
Age, y, median (range)	51 (19–82)
Male	100 (69)
Race and ethnicity	
White, not Hispanic	56 (39)
White Hispanic	39 (27)
Black	25 (17)
Asian/Pacific Islander	5 (3)
Others	20 (14)
Chest imaging findings	
Focal consolidation	25 (17)
Multilobar consolidations/nodules	118 (81)
Cavitary lung lesions	36 (25)
Hilar lymphadenopathy	46 (32)
Pleural involvement	41 (28)
Extrapulmonary^a^	41 (28)
Other infections^b^	73 (50)
Comorbidities	
Immunocompromised^c^	36 (25)
Diabetes mellitus	51 (35)
Chronic kidney disease	17 (12)
Cirrhosis	13 (9)
Diagnosis before intensive care unit^d^	62 (43)
Received anti-*Coccidioides* treatment	132 (91)
Fluconazole	110 (76)
Posaconazole	7 (5)
Voriconazole	32 (22)
Isavuconazole	24 (17)
Liposomal amphotericin B	50 (34)
Death during hospitalization	70 (48)

^a^38 involved >1 organ in addition to the lungs, and 3 involved only musculoskeletal tissue.

^b^62 with bacterial infection, 30 with respiratory viral infection (COVID-19, n = 28), and 11 with other fungal infection.

^c^13, advanced HIV; 8, autoimmune/rheumatologic disorder with steroids and/or other immunosuppressants; 6, solid organ transplant; 5, hematologic malignancies; and 4, solid tumor with chemotherapy.

^d^Diagnosis before intensive care unit with any positive serology or first positive fungal culture.

Most pulmonary infections were multilobar (n = 118, 81%). There were 127 patients with available information regarding the type and duration of symptoms prior to admission, with most endorsing respiratory symptoms (n = 103, 81%) and some reporting constitutional symptoms (n = 50, 39%), such as headache, fever, and myalgia/arthralgia. Ninety-four (74%) experienced symptom onset within 1 week before presentation, and the remaining had symptoms for >2 weeks. The majority of patients (91%) received antifungal therapy during hospitalization. There were 70 (48%) deaths during hospitalization, and 11 of 13 (85%) patients who did not receive antifungal therapy died.

There were 130 cases with available *Coccidioides* serology results, of which 96 (74%) had positive test results; of those, 70 were available prior to the final fungal culture result. The remaining clinical characteristics can be reviewed in Table 1. Complement fixation titers were available for 99 cases (median, 1:16; range, <1:2 to ≥1:28). Complement fixation titers per the patient's mortality outcome were a median 1:8 (range, <1:2 to ≥1:28) for patients who survived vs 1:16 (range, <1:2 to ≥1:28; *P* = .054) for those who died.

### Risk Factors Analysis

The median hospital and ICU lengths of stay were 17 days (range, 2–202) and 12 days (range, 1–73), respectively. As seen in Table 2, older age (≥60 years), liver cirrhosis, and need for mechanical ventilation or vasopressor support use had statistically significant associations with inpatient all-cause mortality. Previous knowledge of coccidioidomycosis diagnosis before ICU admission, use of antifungal therapy before ICU admission, and any use of antifungal therapy had a statistically significant association with inpatient survival. However, male gender, extrapulmonary coccidioidomycosis, diabetes mellitus, chronic kidney disease, and the presence of other infections did not have a statistically significant association with mortality outcome in the univariate logistics regression and were not included in the multivariate model. There were 28 cases of COVID-19, for which the mortality outcome was 54% (n = 15) vs 47% (n = 55) for those without (*P* = .53). We evaluated the effect of pre-ICU antifungal therapy on all-cause mortality using a multivariate-adjusted logistic regression model. Pre-ICU antifungal therapy did not achieve statistical significance (odds ratio [OR], 0.5; 95% CI, .2−1.2) after adjusting for age ≥60 years (OR, 7; 95% CI, 2.6–18.8), liver cirrhosis (OR, 13.1; 95% CI, 1.6–108.8), and mechanical ventilation or vasopressor support use (OR, 15.4; 95% CI, 3.9–59.6), whereas the other factors were statistically associated with all-cause mortality in the model (Table 3).

**Table 2.** Risk Factors Associated With All-Cause Mortality During a Hospital Stay

	Alive (n = 75)	Dead (n = 70)	*P* Value
Age ≥60 y	12 (16)	31 (44)	**<**.**001**
Male	23 (31)	22 (31)	.92
Race and ethnicity			.83
White, not Hispanic	31 (41)	25 (36)	
White Hispanic	18 (24)	21 (30)	
Black	14 (19)	11 (16)	
Asian/Pacific Islander	3 (4)	2 (3)	
Others	9 (12)	11 (16)	
Immunocompromised	21 (28)	15 (21)	.36
Diabetes mellitus	24 (32)	27 (39)	.41
Chronic kidney disease	9 (12)	8 (11)	.92
Extrapulmonary coccidioidomycosis	19 (25)	22 (31)	.42
Cirrhosis	1 (1)	12 (17)	.**001**
Other infections	35 (47)	38 (54)	.36
Mechanical ventilation or vasopressor support	47 (63)	66 (94)	**<**.**001**
Coccidioidomycosis			
History before admission	24 (32)	10 (14)	.**012**
Diagnosis before intensive care unit	43 (57)	19 (27)	**<**.**001**
Treatment before intensive care unit	44 (59)	25 (26)	.**006**
Treated for coccidioidomycosis	73 (97)	59 (84)	.**006**
Antifungal therapy			
Fluconazole	69 (92)	41 (59)	<.001
Posaconazole	5 (7)	2 (3)	.29
Voriconazole	18 (24)	14 (20)	.56
Isavuconazole	11 (15)	13 (19)	.53
Liposomal amphotericin B	25 (33)	25 (36)	.76

Data are presented as No. (%). Bold indicates *P* < .05.

**Table 3.** Multivariate Survival Risk Analysis Based on a Logistic Regression Model

	Unadjusted Model		Adjusted Model^a^	
	Odds Ratio (95% CI)	*P* Value	Odds Ratio (95% CI)	*P* Value
Age ≥60 y	4.2 (1.9–9.1)	**<**.**001**	7.0 (2.6–18.8)	**<**.**001**
Cirrhosis	15.3 (1.9–121.2)	.**01**	13.1 (1.6–108.8)	.**02**
Mechanical ventilation or vasopressor support	9.8 (3.2–29.9)	**<**.**001**	15.4 (3.9–59.6)	**<**.**001**
Coccidioidomycosis treatment before intensive care unit	0.4 (.2–.8)	.**006**	0.5 (.2–1.2)	.11

Bold indicates *P* < .05.

^a^The adjusted model included statistically significant odds ratios in the univariate analysis in Table 2.

## DISCUSSION

Our study demonstrates a high mortality rate (48%) among patients who are critically ill with severe coccidioidomycosis. We identified possible factors associated with increased all-cause mortality, such as older age, liver cirrhosis, and mechanical ventilation or vasopressor support. While early coccidioidomycosis diagnosis and initiation of therapy before ICU admission may have a protective effect against all-cause mortality, this finding did not remain significant after adjusting for other aforementioned risk factors.

Severe coccidioidomycosis, due to pulmonary and disseminated disease, is associated with increased mortality and, depending on the host factors, can range broadly between 5% and 75% [13, 16, 20]. Data on patients who are critically ill with coccidioidomycosis outcomes have been limited, but in 2 previous studies, the mortality rate ranged between 15% and 33% [18, 19]. However, these previous studies mainly included patients with serologic diagnosis and pulmonary involvement. In contrast, our study had a mortality rate of 48%, but we included only culture-proven coccidioidomycosis cases with a variety of disease presentations. These presentations featured 28% with extrapulmonary disease, which may explain the higher mortality rate.

Risk factors for severe coccidioidomycosis, such as diabetes mellitus, immunocompromising conditions, and male gender, were previously recognized [13, 21, 22]. However, among our cohort of patients with coccidioidomycosis who were critically ill, we did not observe a statistically significant all-cause mortality association with the aforementioned risk factors. Other risk factors for severe disease are race and ethnicity, as Black race and Filipino ethnicity have been associated with a higher risk for severe coccidioidomycosis and worse outcomes [22]. While the patients in our study did not have granular information regarding their ethnicity, we did not find a statistical association between race and all-cause mortality. It is noteworthy to mention that our cohort constituted 17% of those who identified as Black, in contrast to 5.5% of Arizona's population [23], which may be partially explained by the racial disparities in accessing health care, leading to the increased risk of coccidioidomycosis complications and severe sepsis among Black people [24]. Yet, such a conclusion is limited because of the absence of data analyzing the effect of race on ICU admission and other diseases vs coccidioidomycosis. It is worth noting that social determinants of health were suggested before as a potential cause of delayed diagnosis and severe infections [25]. More studies addressing racial disparities and access to health care among Black patients with coccidioidomycosis are needed in the endemic region to improve early diagnosis and treatment.

Similar to previous reports of older age as a risk factor for coccidioidomycosis-associated mortality, we found that age ≥60 years was associated with higher odds for all-cause mortality [26]. Also, most of our critically ill cohort had concomitant infections besides coccidioidomycosis, which increased the severity of their outcomes. However, this risk of concomitant infection did not have a statistically significant association with all-cause mortality. COVID-19 was not statistically associated with increased mortality, but it is worth noting that we do not have detailed information regarding the severity of COVID-19 cases. COVID-19 infection in severe ICU cases generally receives high steroid therapy, which might have contributed to the reactivation of coccidioidomycosis in some of those cases, but we do not have data to explore this hypothesis. Another risk factor is the immunocompromising condition, which is associated with severe coccidioidomycosis and increased mortality among patients with transplants. We had 28% of cases with extrapulmonary involvement, but we did not find a statistically significant association between mortality and extrapulmonary coccidioidomycosis. This relatively high number of extrapulmonary coccidioidomycosis cases is likely due to our including a critically ill cohort with coccidioidomycosis presentation. It was previously reported that patients with allogeneic hematopoietic stem cell transplantation and severe disease have a mortality rate of about 50% [20]. Yet, in our cohort, we did not find a statistically significant association between all-cause mortality and immunocompromising conditions. This observation is likely secondary to the heterogeneity of the immunosuppressed conditions in the cohort, which also featured 6 solid organ transplant recipients who were receiving prolonged coccidioidomycosis prophylaxis. Nevertheless, it is important to note that without proper diagnosis and appropriate therapy, severe coccidioidomycosis can be fatal among patients who are highly immunocompromised.

Evidence supporting the association of severe coccidioidomycosis and liver cirrhosis is not well established. However, recent case series have reported severe and disseminated diseases among patients with end-stage liver disease, suggesting a possible association between severe coccidioidomycosis and cirrhosis [8, 27, 28]. This may be related to cirrhosis-associated immune dysfunction, which can generally increase the risk for bacterial and fungal infections [29]. Such immune dysfunction has been characterized by a pattern of immune dysregulation extending from reduced phagocytosis and B- and T-cell functions to decreased production of tumor necrosis factor α [30, 31], which have been described as possible risk factors for severe infections, including coccidioidomycosis. Indeed, patients with acute and chronic liver failure are predisposed to different invasive fungal infections, ranging from cryptococcosis to candidiasis and aspergillosis [32]. Beyond severe coccidioidomycosis, liver cirrhosis with sepsis tends to have a poor prognosis, as such patients have an increased risk of mortality of up to 75% [33]. In our cohort, most patients with a diagnosis of cirrhosis (12/13) died during their ICU stay. Yet, further studies on the role of immune system regulation in coccidioidomycosis and the immunologic role of the liver function may elucidate the basis for this increased risk of severe disease in patients with cirrhosis.

We explored the role of antifungal use on all-cause mortality among our cohort, as we noted that 11 of the 13 who did not receive any antifungal therapy died. It was previously shown that antifungal administration before hospitalization could result in a lower risk of severe disease [13].

Therefore, we investigated the role of pre-ICU antifungal administration on the all-cause mortality associated with coccidioidomycosis, and in the univariate analysis, pre-ICU antifungal therapy had a statistically significant association with reduced all-cause mortality. However, after adjusting for underlying risk factors of liver cirrhosis, mechanical ventilation or intravenous pressors, and older age, the association between pre-ICU antifungal therapy and all-cause mortality was not statistically significant. One possible explanation is that patients who required ICU hospitalization had already progressed to severe disease and the antifungal therapy effect may have been minimal vs a more efficacious approach of earlier administration of antifungal therapy. Moreover, our study did not evaluate the effect of antifungal therapy timing for the period from developing coccidioidomycosis to ICU admission, which can influence the mortality outcome among patients with coccidioidomycosis. We also did not evaluate the effect of different antifungal therapies on the mortality outcome, given the heterogeneity of antifungal use and confounding by indication. Therefore, it remains plausible that early diagnosis with prompt antifungal therapy initiation before the advancement of the disease to a critical stage might substantially reduce mortality associated with coccidioidomycosis.

Our study strengths include the large sample size and inclusion of only culture-positive, proven cases of coccidioidomycosis. Specifically, we included only culture-proven cases admitted to the ICU, which includes only definitive diagnoses, consistent with the 2020 “Revision and Update of the Consensus Definitions of Invasive Fungal Disease From the European Organization for Research and Treatment of Cancer and the Mycoses Study Group Education and Research Consortium” [10]. Moreover, we evaluated different risk factors and used univariate and multivariate analyses using logistic regression to evaluate the risk factors related to the outcome of all-cause mortality.

Our study limitations include the retrospective design and related selection bias. Most cases were evaluated for all-cause mortality during the hospital stay, which can result in biases involving loss of follow-up and analyzing specific etiology leading to the patient's death. As such, our study did not measure attributable mortality due to coccidioidomycosis, and we could not provide information on each patient's death etiology because of the multiple underlying comorbidities that patients had, including COVID-19. However, including only culture-proven coccidioidomycosis cases suggests that *Coccidioides* species was active and likely contributed to the patients’ ICU hospitalization and outcome. Another limitation of the study is that we do not have information on or an analysis of coccidioidomycosis prior to ICU admission, which could have helped inform the course of coccidioidomycosis that resulted in the ICU admission and mortality outcome. We were also not able to evaluate antifungal efficacy, including the type of antifungal therapy, because the majority of patients received different antifungals during their hospital stay and a small sample received nonfluconazole antifungal therapy. We do not have granular information regarding the type of concomitant infections that can affect all-cause mortality. Last, by including only culture-positive cases, we are missing cases with positive pathology or serology.

In conclusion, the mortality among patients who were critically ill with culture-proven coccidioidomycosis was high (48%). Among this population with multiple coinfections, antifungal therapy did not reduce all-cause mortality after accounting for several risk factors. However, future studies should investigate the role of early diagnosis and therapy in patients at high risk before clinical deterioration to a critical condition.

## Note

***Potential conflict of interests***. All authors: No reported conflicts.
